# Prevalence and lifestyle-associated risk factors of metabolic syndrome among commercial motor vehicle drivers in a metropolitan city in Ghana

**DOI:** 10.11604/pamj.2020.36.136.16861

**Published:** 2020-06-29

**Authors:** Collins Afriyie Appiah, Edward Opoku Afriyie, Frank Ekow Atta Hayford, Emmanuel Frimpong

**Affiliations:** 1 Department of Biochemistry and Biotechnology (Human Nutrition and Dietetics Unit), Faculty of Biosciences, College of Science, Kwame Nkrumah University of Science and Technology, Kumasi, Ghana,; 2 Department of Nutrition and Dietetics, School of Biomedical and Allied Health Sciences, College of Health Sciences, University of Ghana, Accra, Ghana,; 3 Centre of Excellence for Nutrition, Faculty of Health Sciences, North West University, Potchefstroom Campus, Potchefstroom, South Africa

**Keywords:** Metabolic syndrome, overweight/obesity, diabetes, hypertension, dyslipidemia, lifestyle-related behaviors, commercial motor vehicle drivers

## Abstract

**Introduction:**

commercial motor vehicle drivers are at risk of metabolic syndrome (MetS) due to the nature of their work as they tend to go to work early, work for more hours, have irregular dietary habits and patterns, have little sleep and live sedentary lifestyle. The study sought to determine the prevalence and lifestyle-related risk factors of MetS among commercial taxi drivers around Kwame Nkrumah University of Science and Technology (KNUST) campus, in the Kumasi metropolis, Ghana.

**Methods:**

a cross-sectional survey was conducted among 100 commercial taxi drivers in 3 selected taxi ranks around KNUST campus. Fasting blood lipid and fasting blood glucose levels, blood pressure and anthropometric characteristics were determined using WHO and NCEP-ATP III criteria. Lifestyle-related risk factors of MetS were assessed using a semi-structured questionnaire and dietary pattern was assessed using food frequency questionnaire. Bivariate analysis and linear correlation were used to determine the relationship between lifestyle practices and MetS.

**Results:**

the prevalence of diabetes, high blood pressure, dyslipidemia, overweight and obesity were 12%, 63%, 40%, 32% and 13% respectively. The prevalence of MetS was 5% according to NCEP-ATP III (2005) criteria. The lifestyle behaviours of the drivers were, alcohol intake, irregular dietary pattern, long working hours, lack of exercise and tiredness due to driving. Tobacco use (R = 0.405, p = 0.041) and time of supper (R = 0.931, p = 0.047) were related with MetS among the participants.

**Conclusion:**

though prevalence of MetS (5%) was low among the drivers, the need for intervention to promote positive lifestyle change and curb the high prevalence of overweight/obesity, diabetes, high blood pressure and dyslipidemia is necessary to improve the health of the drivers and the safety of passengers.

## Introduction

Metabolic syndrome (MetS) which has been described as insulin resistance, deadly quartet and syndrome X, is a cluster of risk factors that raises the risk of heart diseases, stroke and other health disorders [1]. It is diagnosed when any three of the five risk factors which include insulin resistance, low levels of high density lipoprotein (HDL) cholesterol, high levels of triglycerides, hypertension and central obesity (large waist circumference) are present [2]. Globally, 20-30% of adults have MetS which relies on the region (rural or urban setting) and the definition of MetS used [3-5]. The prevalence of MetS among adult Ghanaians was 12.4% and 21.2% using NCEP-ATP III (2005) and IDF (2005) criteria respectively (2015) [6]. Certain jobs predispose workers to health hazards which can be mechanical, physical and chemical hazards which commercial taxi drivers are not exception [7]. Commercial taxi drivers are professional drivers that mostly drive for a shorter distance [8]. Most people in urban areas rely on commercial taxi to commute from place to place. Commercial taxi drivers are important players in the transportation nexus of cities all over the world including Ghana. Studies have indicated that lifestyle practices such as excessive alcohol consumption, smoking, physical inactivity, poor dietary habits and patterns and stress have impact on the development of MetS [9-11]. Studies have shown that commercial drivers are prone to lifestyle-related risk factors of MetS due the nature of their work as they tend to go to work early, work for long hours, have irregular dietary habit and pattern and have little sleep [10-12]. These tend to pose a threat to the health of the driver and the safety of the passengers. In the case of commercial taxi drivers in Kumasi there is paucity of data on the prevalence and lifestyle-related risk factors of MetS, hence this study sought to determine the prevalence and lifestyle-related risk factors of MetS among commercial taxi drivers around KNUST campus, Kumasi.

## Methods

**Subjects:** in this cross-sectional survey, one hundred (100) commercial taxi drivers aged 20 years and above were recruited from 3 selected taxi ranks (stations) around KNUST campus, Kumasi metropolis. Participants were recruited using convenient sampling technique. All the subjects were males due to the nature of work as commercial motor vehicle drivers are predominantly males. Drivers who decline to participate in the study were excluded.

**Data collection:** the participants underwent 12-hour overnight fasting after which blood sample was collected for fasting blood lipid and fasting blood glucose levels determination. Blood pressure and anthropometric characteristics (weight, height, BMI and waist circumference) were determined. Lifestyle-related risk factors of MetS were assessed using a semi-structured questionnaire and dietary pattern was assessed using a modified National Health and Nutritional Examination Survey (NHANES) food frequency questionnaire.

**Ethical approval:** written informed consent was obtained from the participants. Permission was sought from the Committee on Human Research, Publications and Ethics of Kwame Nkrumah University of Science and Technology, School of Medical Sciences and Komfo Anokye Teaching Hospital Kumasi, Ghana prior to all research procedures (ethical identification number: CHRPE/AP/026/18).

**Statistical analysis:** data collected were entered into IBM Statistical Package for Social Sciences (SPSS) version 20 for analysis. Categorical variables were presented as frequencies and percentages whilst continuous variables were presented as means and standard deviations. ANOVA was also used to determine the differences between more than two means. Scree plot after principal component analysis was used to analyze food frequency data. Bivariate correlation was used to determine the correlation between lifestyle practices and MetS. Linear regression was used to determine the relationship between lifestyle practices and MetS. The level of statistical significance was set at p < 0.05.

## Results

**Figure 1:**
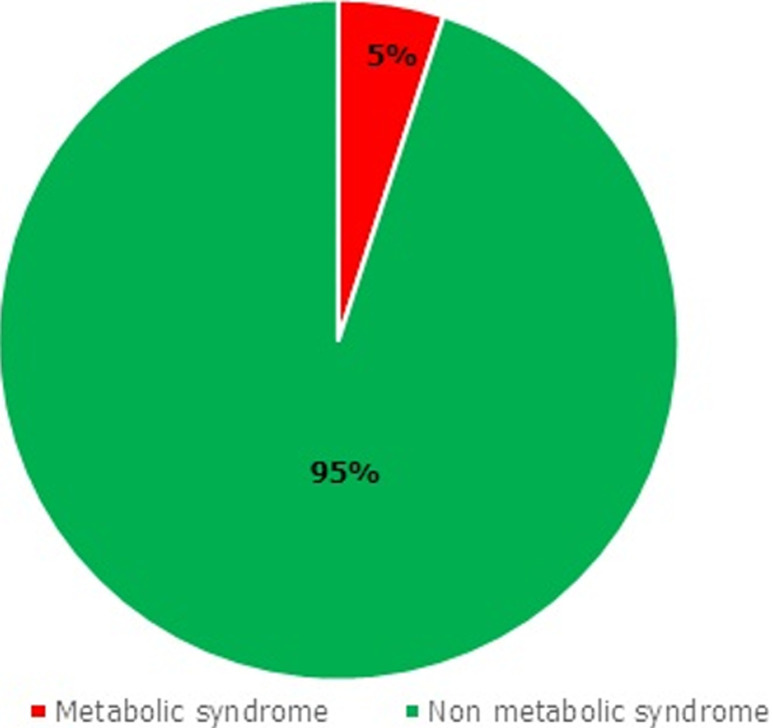
prevalence of metabolic syndrome among the participants

**Figure 2:**
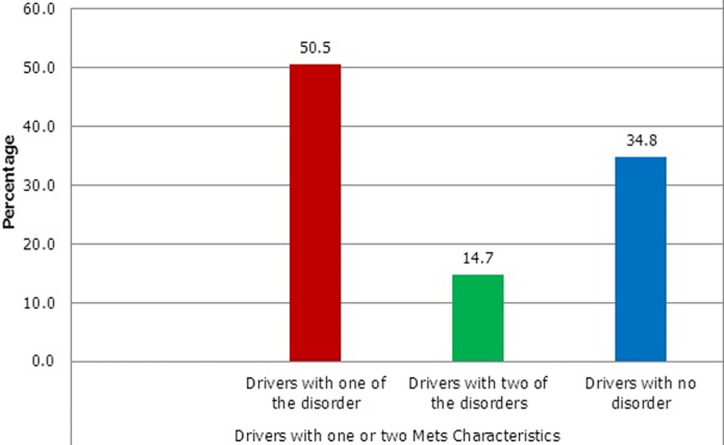
drivers with two or one of the metabolic syndrome parameters

The socio-demographic data on the study participants are shown in Table 1. The mean age of the participants was found to be 41 ± 8.9 years with a minimum age of 25 years and a maximum age of 69 years. In terms of educational background, majority of the participants (98%) had some level of formal education. Most of the participants (67%) had their education up to the junior high school level. Moreover, most of the participants (51%) had driven commercial taxis up to 10 years. The prevalence of overweight, obese high blood pressure, dyslipidemia and diabetes among the participants are indicated in Table 2. Majority of the drivers, (52%) had normal BMI, 32% were overweight and 10% were obese. According to the waist circumference, 13% were obese. Sixty-three (63%) percentage of the participants had high blood pressure. Moreover, 40% had dyslipidemia. With respect to fasting blood glucose levels, 12% were found to be hyperglycemic. Figure 1 shows the prevalence of MetS among the participants. The prevalence of MetS among the drivers was 5% based on the NCEP-ATPII (2005) criteria. Shown in Figure 2 is the participant with one or two of the MetS characteristics. From Figure 2, 50.5% and 14.7% of the drivers were also found to have one or two of the diagnostic criteria for MetS respectively, which include diabetes, low HDL, high blood pressure, high triglycerides (TGs) and central obesity. Lifestyle characteristics of the drivers are shown in Table 3. Majority of the drivers (40%) become very tired whilst 30% often become tired as a result of driving. Most of the drivers (72%) did not engage themselves in any exercise. From this study it was found out that 60% of the participants, watch television when they were not working, 11% play in door games such as ludo and “oware” (pit and pebble) and 19% tend to sleep when not at work.

**Table 1:** socio-demographic characteristics of the participants (N = 100)

Variables	Frequency	Percentage (%)
**Age (Years)**		
20-29	8	8.0
30-39	46	46.0
40-49	28	28.0
50-59	15	15.0
≥60	3	3.0
**Educational level**		
Primary	9	9.0
JHS	62	62.0
SHS	25	25.0
Tertiary	2	2.0
None	2	2.0
**Years of driving commercial taxi**		
≤10 years	51	51.0
11-20 years	38	38.0
21-30 years	9	9.0
≥31 years	2	2.0

JHS-Junior high school education; SHS-Senior high education; Tertiary-University education, polytechnic education, nursing and teacher training education; N-total number of participants

**Table 2:** prevalence of overweight, obesity, blood pressure, dyslipidemia and diabetes (N = 100)

Variables	Frequency	Percentage (%)	Mean ± SD
**BMI**			24.4±4.0
Underweight	6	6.0	
Normal range	52	52.0	
Overweight	32	32.0	
Obese	10	10.0	
**Waist Circumference**			34.6±4.9
Normal	87	87.0	
Obese	13	13.0	
**Blood pressure (BP)**			131±18.5/87±13.9
Normal BP	37	37.0	
High BP	63	63.0	
**Dyslipidaemia**			
Normal Cholesterol levels	60	60.0	
Dyslipidaemia	40	40.0	
**Fasting blood glucose (FBG)**			5.0±1.7
Normal	88	88.0	
Hyperglycaemia	12	12.0	

BMI categorization is by WHO (1998) criteria and all the other parameters are categorized by NCEP-ATP III (2005) criteria.

**Table 3:** lifestyle characteristics of the participants

Variables	Frequency	Percentage (%)
**Tiredness because of driving**		
Never	4	4
Sometimes	23	23
Often	33	33
Very often	40	40
**Sleeplessness because of driving**		
Never	53	53
Sometimes	27	27
Often	8	8
Very often	12	12
**Activity done when not driving**		
Watching TV	60	60
Playing in-door games	11	11
Visiting friends and family members	5	5
Attending social functions	5	5
Sleeping	19	19
**Alcohol intake**		
Yes	43	43
No	57	57
**Tobacco use**		
Yes	5	5
No	95	95
**Sleeping hours**		
*Normal sleep	43	43
**Less sleep	57	57
**Working hours per day**		
≈Long working hours	100	100
**Not eating well because of driving**		
Never	46	46
Sometimes	22	22
Often	20	20
Very often	12	12
**Breakfast**		
Yes	19	19
No	81	81
**Eat after work in the evening**		
Yes	64	64
No	36	36
**Time for supper**		
⋅Early	19	19
⋅⋅Late	81	81

* 7-9 hours, **less than 7 hours, ≈more than 10 working hours, ⋅less than 7:30pm, ⋅⋅more than 8pm

Forty-seven (47%) participants suffer sleeplessness due the driving. Forty-three (43%) of the participants take alcohol. On the contrary, tobacco use was relatively low among the participants only 5% using tobacco. With respect to their sleeping pattern most of the drivers 57% have little sleep (sleeping hours less than 7 hours). In addition, all the drivers were found to work for 10 hours or more a day. Per the number of meals/day of the participants, majority (55%) eat thrice a day whilst 43% eat twice times a day. Most (81%) of the participants skip breakfast. With respect to the drivers eating in the evening when they return home, 64% eat when they arrived home. Moreover, it was found out from the study that majority of the drivers 81% eat late (› 7: 30 pm) in the evening. The mean time that these drivers usually take super was 8: 00pm ± 1.82 with earliest and latest eating times being 4: 00 pm and 12: 00 am (midnight) respectively. Table 4 and Table 4 (suite) indicates dietary pattern of food groups consumed by participants. Five patterns were generated from scree plot after principal component analysis of food frequency data. Pattern 1 had the most consumed pattern, followed by pattern 2 and pattern 5 had the least consumed foods. The patterns were grouped according to correlation coefficient factor ≥ 0.3 for positive and negative values. The principal component analysis showed five components with eigen values exceeding 1; explained as percentage of variances: 15.2%, 5.8%, 5.1%, 4.9% and 3.9% respectively. Table 5 shows the relationship between lifestyle characteristics and systolic blood pressure, diastolic blood pressure, dyslipidemia and MetS. Age, smoking, physical activity, years of driving, sleeping hours, stress level and time for supper showed a relationship with SBP. As shown in the Table 5, age, years of driving and sleeping hours were strongly positively correlated to diastolic blood pressure (DBP). On the contrary, exercise was negatively correlated with DBP. Age, hours of sleep and years of driving showed a strong correlation (R = 1.11; R = 0.61; R = 0.84) with DBP respectively. Physical activity showed a relationship with dyslipidemia and was strongly related with dyslipidemia. Physical activity was strongly correlated with TC, triglyceride and LDL (R = -0.97; R = -0.99; R = -0.59) respectively. Tobacco use (smoking) and time for supper were related with MetS. Tobacco use and time of supper were strongly related to MetS (R = 0.405; p = 0.041; R = 0.931; p = 0.047).

**Table 4:** dietary patterns of the participants

Dietary pattern	Pattern 1	Pattern 2	Pattern 3	Pattern 4	Pattern 5
**% Variances**	15.2	5.8	5.1	4.9	3.9
**Food sources**	All Varied food groups except plant oils and tiger nut	Non-fruits, non-oils and nuts, non-fish, meat and poultry, spring roll, rock buns, and processed foods	Non-fruits, non-vegetables, non-oils and nut, non-fish and meat, soft drinks, fried rice	Citrus fruits, garden eggs, cabbage stew, non-fish and meat, polished rice and salad cream	Tomatoes, tomatoes stew, plant oils, non-meat
**Fruits**					
Watermelon	0.394				0.363
Banana	0.325				
Citrus	0.332			0.300	0.375
Mango	0.464		-0.387		
Pawpaw	0.380		-0.309		
Pineapple	0.362				
Apple	0.356				
**Vegetables**					
Tomatoes		0.360			0.411
Garden eggs	0.372	0.300		0.340	
Lettuce					
"Kontomire"	0.317				
Carrot	0.462				
Cabbage	0.328				
**Oil**					
Plant oils					0.369
**Fishes**					
Salmon					
Herrings					-0.316
Tuna	0.544				
Oysters	0.410				
**Meat and poultry**					
Beef	0.551			-0.510	
Pork	0.341			-0.305	

**Table 4 (suite):** dietary patterns of the participants

Food sources	Pattern 1	Pattern 2	Pattern 3	Pattern 4	Pattern 5
Meat				-0.358	
Chicken					
Corned beef	0.429			-0.551	
Bush meat	0.473			-0.535	
**Carbohydrates**					
Oats	0.370				
Wheat	0.313	0.317			
"Kenkey"			-0.431		
"Banku"	0.396				
Rice	0.344				
Sugar	0.455				
"Tz"	0.380				
Bread	0.453				-0.378
Plantain			0.515		
Cocoyam	0.419			-0.387	
Yam					
Cassava			0.746		
**Snacks**					
Rock buns	0.591	-0.305			
Spring rolls	0.588	-0.427			
Meat pie	0.497				
Chips	0.681				
Roasted nuts	0.516				-0.327
**Processed foods**					
Soy sauce	0.455	-0.710			
Mayonnaise	0.457	-0.703			
Salad cream	0.489	-0.486		0.356	
Beverages					-0.310
Soft drinks			0.399		

Foods sources with asterisks are local foods eaten in Ghana.

**Table 5:** relationship between systolic blood pressure, diastolic blood pressure, dyslipidemia, MetS and lifestyle behaviours

Lifestyle characteristics	*Systolic Blood Pressure			*Diastolic Blood Pressure			*Dyslipidemia			*†MetS		
	B (95% CI)	R	p-value	B (95% CI)	R	p-value	B (95% CI)	R	p-value	B (95% CI)	R	p-value
**(Constant)**	153.1		0.007	128.9		0.026	1.824		0.015	0.67		0.363
**Age**	30	1.41	0.001	18.5	1.11	0.032	0.016	0.306	0.359	0.016	0.586	0.393
**Years of driving**	30.6	0.99	0.001	20.3	0.84	0.001	-0.125	-0.180	0.231	-0.02	-0.052	0.836
**Sleeping hours**	-10	-0.68	0.001	7.1	0.61	0.021	0.017	0.049	0.664	0.181	0.351	0.26
**Sleeplessness**	0.131	0.268	0.017	0.186	0.381	0.000	0.029	0.065	0.560	0.071	0.328	0.067
**Tiredness**	0.059	0.104	0.312	0.058	0.102	0.286	0.012	0.023	0.828	0.007	0.025	0.886
**Time of supper**	10.3	0.98	0.001	-0.055	-0.067	0.776	0.161	0.216	0.382	0.357	0.931	0.047
**Tobacco use**	41	0.52	0.001	21.4	0.35	0.042	-0.127	-0.060	0.574	0.405	0.405	0.041
**Exercise**	-62.3	-0.9	0.001	-45.2	-0.84	0.002	-0.278	-0.272	0.012	-0.12	-0.205	0.316
**Alcohol**	0.140	0.140	0.172	0.092	0.091	0.348	-0.025	-0.027	0.800	-0.016	0.033	0.135

R=correlation coefficient (standardized coefficient), *dependent=variable, B=unstandardized coefficient, p-values are statistically significant at p<0.05 †MetS= Metabolic syndrome

## Discussion

In this present study, the mean age of the drivers was found to be 41 ± 8.9 years. According to works done on commercial drivers by Abban [10], Achulo *et al*. [12] and Asiamah *et al*. [13] the average age of commercial drivers were 40.78 ± 8.6, 38.8 ± 13.7, 41.2 ± 8.6 and 41.2 ± 11.7 years respectively. Educational background of commercial drivers has been found to differ from drivers at different places which mostly comprises of school dropouts, technical and secondary school leavers, and a few tertiary graduates [10]. In this work, the educational profile of the commercial taxi drivers was such that 2%, 9%, 62%, 28% and 2% had no formal education, primary education, basic education (junior high school leavers), secondary education and tertiary education respectively similar to the trend reported by Achulo *et al*. [12]. Majority of the drivers opt for this occupation because they do not have the qualification to secure jobs of their interest since due to limited education and financial difficulty/difficulty accessing credit facilities to start their own enterprises. As a result, majority drive other people´s cars and the demand from these owners make them work for long hours (more than 10 hours a day) to obtain the sales per day demanded from the owners. Inconsistent with these findings on the educational profile of the participants, 16%, 60% and 23% of the drivers in Accra had no formal education, primary education and secondary education respectively [13]. Moreover, 1.7%, 37.9%, 58.6% and 1.7% of the drivers in Accra had no formal education, primary education, secondary education and tertiary education respectively [12]. The nature of work of commercial drivers as they tend to go to work early, return late, sit and work for long hours tend to make them have irregular dietary pattern and habits [14]. As a result, they tend to consume foods from fast food services and food vendors which are low in fruits and vegetables, high in calories and salt [15]. From this work, the drivers were found not consuming fruits regularly similar to other researches. A study among Hon Kong drivers showed that 38.8% of the drivers take less than one fruit per day [9]. About half (54%) of them were also found to consume foods from fast food joints and food vendors [9]. This habit is associated with overweight and obesity which are risk factors of MetS. Eating more fruits and vegetables also lower the risk of coronary heart diseases (CHDs) [16].

In this present work, it was found that majority of the drivers depend on fast foods and foods from vendors as most of the drivers (81%) skip breakfast in their homes. This is because they are required to go to work early in order to meet their daily requirements. In addition, they mostly take lunch also from these same vendors. Again about 36% take supper from these same food sources which could lead to increased salt and fat intake as commercial vended foods tend to be high in salt and fat. Furthermore, due to their work demands, they return home late (7pm-12am) and hence eat late in the evening usually foods which are all high calorie foods. Moreover, they tend to go to bed right away since they need to go to work early the next morning. These practices all together could contribute to development of overweight and obesity, dyslipidemia, and hypertension which are hallmarks components of MetS [1]. These findings are also in line with other findings [10,12,17]. Stress occurs when environmental demands exceed the adaptive capability of the individuals [18]. This can be physiological, psychological and behavioural [19]. Studies have shown that stress has an effect on the health of an individual and the effect is dependent on the degree of the stress be it acute, or high stress [20]. Works involving long working hours and less sleep have been found to increase stress levels and this increases the risk of cardiovascular diseases (CVDs). A research done in Japan revealed that workers who work for more than 10 hours a day increase their risk of developing CVDs by a factor of 2.4 more than workers who work within 7-9 hours [21]. From this present study, it was found that all the drivers work for more than 10 hours a day and had little sleep (less than 7-9 hours sleep a day). In males of Taiwan, 22% of the drivers work for than 10 hours a day [21]. Again, most of the drivers reported been tired after their days´ activities. These behaviours could contribute to stress which has the tendency of increasing the risk of developing cardiovascular diseases. This could be the reason of the high prevalence of high blood pressure reported among the participants. This gives an indication that commercial drivers tend to be stressed from the nature of their work. Most drivers consume alcohol because it is seen as a means of relaxation after a hard day´s work, appetite enhancement and as part of social events [10]. Alcohol when excessively consumed has been found to accumulate triglycerides, increase blood pressure and increase calorie intake [22].

This high calorie intake can contribute to the development of overweight, obesity and dyslipidemia which increase the risk of developing MetS [23]. Findings of this study show that 43% of the drivers were found to consume alcohol with the majority taking beer. This habit could be the reason for the increase in prevalence of dyslipidemia reported among the drivers and could lead to liver damage if not addressed [22]. In comparison to the works by Abban [10] and Asiamah *et al*. [13], they all recorded that 50% of the drivers consume alcohol. However, Asiamah *et al*. [13] found that majority were consuming spirit rather than beer which was observed in this present study. This shows that the drivers consume more alcohol which could increase their chances of developing dyslipidemia, obesity, high blood pressure and other health disorders. Enough physical activity has been found to have a good health benefit in individuals and has an independent direct effect of lowering the risks of cardiovascular diseases (CVDs) [24]. This study found that majority of the drivers did not engage in any form of exercise. Furthermore, with respect to the activity done when not driving, 60% watch television and 19% sleep which gives an indication of sedentariness among the drivers. This sedentary lifestyle could partly explain the reason the high prevalence of high blood pressure, dyslipidemia and the prevalence of the other MetS diagnostic parameters among the drivers. Similar trend of physical inactivity was reported in other works among commercial drivers [9,10,12]. Smoking, the second leading cause of CVDs after hypertension [15] was less among the drivers as 5% smoke. Even though the prevalence was relatively less, smoking has the tendency to cause high blood pressure and other health problems including MetS [22] and this demands the attention of health professionals for strategies to reduce smoking among drivers. Increasing levels of LDL cholesterol, TGs and decreasing levels HDL cholesterol contribute to the formation of plagues in the blood vessels increasing the chance of developing high blood pressure and its related cardiovascular diseases [25]. Findings of this present study shows that even though the drivers reported practicing similar lifestyle-related risk factors, all the drivers (100%) had normal levels of HDL cholesterol (› 1.04mm/L). Again, from this study, a few of the drivers (2%) had high levels of TGs. However, high levels of LDL cholesterol levels were recorded (30%) and this contributed to the high prevalence of dyslipidemia (40%) reported. High LDL cholesterol ("bad cholesterol") levels have the potential to cause atherosclerosis. The formation of plaques in the blood vessels can cause high blood pressure and other cardiovascular complications and compromise the quality of health of the drivers. These findings could be attributed to behaviors such as excessive alcohol intake and sedentary lifestyle among the drivers. The prevalence of diabetes, another component of MetS, was found to be 12% among the drivers. This was not consistent with other studies [10].

Abban [10] and Kurosaka [26] recorded 72.7% and 50.9% prevalence of diabetes respectively. The prevalence of diabetes among the drivers could be linked to the high prevalence of obesity which is a risk factor of diabetes [27], physical inactivity and excessive alcohol consumption. Diabetes if not controlled can lead to other health complications such as atherosclerosis, metabolic syndrome, and other cardiovascular events. In 2011, the prevalence of MetS among bus and truck drivers in Iran was found to be 35.9% [14]. This finding was higher than the general prevalence of MetS in male Iranians (22%). This gave an indication that driving as occupation poses an individual to the risk of developing MetS. This was attributed to the poor dietary habits and lifestyle practices of the drivers. Even though some of such behaviours were consistent with the findings of this study, 5% prevalence of MetS was recorded which is lower than that reported in the Iranian study. On the contrary, 50.5% and 14.7% of the drivers in this study had one or two of the diagnostic parameters of Mets respectively. In line with the pathogenesis of MetS, the development of one of the components tend to set the pace for the development of others [1]. This suggests the drivers with one or two MetS components are at risk of developing MetS in the future if no intervention is implemented. These could be attributed to their lifestyle practices, nature of work and its demands. From this study, the older drivers were at risk of high systolic and diastolic blood pressure. Aging by one year increases the risk of developing systolic and diastolic blood pressure (SBP and DBP) by 30% and 18.5% respectively. It has been documented that aging is associated with high blood pressure [28]. Moreover, years of driving was also strongly correlated to SBP and DBP. As the years of being in the driving occupation increases, the probability of developing SBP and DBP is 30.6% and 20.3% respectively. This observation could be as a result of the older drivers being physically inactive for a longer time and exposure to the lifestyle-related risk factors over a long period. Sleeping hours, time of supper, tobacco use and exercise had a strong relation with SBP. As these parameters increase by a point there is a corresponding increase in the chance of developing SBP by 10%, 10.3%, 41% and 62.2% respectively. On the other hand, sleeping hours, tobacco use, and exercise had a strong impact on DBP among the participants. As the drivers continuously involve themselves in these activities, their chances of developing DBP were 7.1%, 21.4% and 45.2% respectively. All these put together could contribute to the high prevalence of high blood pressure among the participants. From the result, exercise was negatively associated with dyslipidemia. As the drivers have little exercise, development of obesity and overweight increase thereby causing abnormal increase in the lipid levels in the blood. This narrows the vessels putting pressure on the heart leading to high blood pressure [29]. In this current work, smoking was associated with the development of MetS. This means that increasing the rate of smoking increases the rate of developing MetS. Oxidative stress generated from smoking has the tendency to cause endothelial injury which could lead to atherosclerosis and hardening and narrowing of blood vessels. This could contribute to the development of high blood pressure a component of MetS [15]. Furthermore, time of supper could also be a contributing factor to the prevalence of MetS among the participants. Eating supper late and sleeping immediately afterwards has the tendency to accumulate fats in the body. During sleep, the body´s basal metabolic rate reduces which will cause the body to store the excess calorie as fats in the adipose tissues mostly in the abdominal region. Increasing abdominal obesity has been linked to diabetes, high blood pressure and dyslipidemia [1]. In view of this increasing the physical activity levels, eating early supper and avoidance of smoking could be useful in preventing high blood pressure, dyslipidemia and the emergence of MetS among the drivers.

## Conclusion

Though the prevalence of metabolic syndrome was low among the drivers in this study, there were high prevalence rates of its components. The lifestyle practices of the participants, if not intervened, could drive an upsurge in metabolic syndrome among them. This calls for urgent intervention strategies to address the lifestyle-related behaviours of the drivers to promote positive lifestyle change and curb the high prevalence of overweight/obesity, diabetes, high blood pressure and dyslipidemia and ultimately improve the health of the drivers and the safety of passengers.

### What is known about this topic

- Commercial drivers are at risk of MetS due to the nature of their work, which puts the health of the drivers and the safety of the passengers at risk;
- The emergence of one of the components of MetS is a risk factor for the others if no intervention is implemented;
- Lifestyle practices such as sedentary lifestyle, smoking, long working hours, excessive alcohol consumption, stress as well as epigenetic and genetic programming are highly associated with the development of MetS.

### What this study adds

- This study suggests that smoking, time of supper and sedentary lifestyle are highly associated with MetS;
- The study also adds that commercial taxi drivers are living sedentary lifestyle, eat late in the evening, rely mostly on fast foods and foods from vendors, work for long hours and consume alcohol;
- This study proposes that strategies against the overweight/obesity, high blood pressure, dyslipidemia and diabetes problems in Ghana should address the nutritional education and lifestyle problems.
